# Time for culture conversion and its associated factors in multidrug-resistant tuberculosis patients at a tertiary level hospital in Peshawar, Pakistan

**DOI:** 10.12669/pjms.38.4.5058

**Published:** 2022

**Authors:** Zafar Iqbal, Mazhar Ali Khan, Aamir Aziz, Syed Muhammad Nasir

**Affiliations:** 1 Zafar Iqbal, Department of Pulmonology, Lady Reading Hospital, Peshawar, Pakistan; 2 Mazhar Ali Khan, Programmatic Management of Drug-Resistant TB Unit, Lady Reading Hospital, Peshawar, Pakistan; 3 Aamir Aziz, Institute of Biological Sciences, Sarhad University of Science and Technology, Peshawar, Pakistan; 4 Syed Muhammad Nasir, Programmatic Management of Drug-Resistant TB Unit, Lady Reading Hospital, Peshawar, Pakistan

**Keywords:** Tuberculosis, MDR-TB, Time to sputum culture conversion

## Abstract

**Objectives:**

This study aimed to assess the time to sputum culture conversion (SCC) and its determinants among multidrug-resistant tuberculosis (MDR-TB) patients.

**Methods:**

This cross-sectional study was conducted from January 2019 to January 2020. A total of 252 MDR-TB patients presenting at a tertiary level teaching hospital in Peshawar, Khyber Pakhtunkhwa (KP), were included. The patient’s demographic and clinical data were collected using a structured questionnaire. Time to SCC was calculated from the initiation of treatment till the patient had two consecutive negative cultures. The Cox proportional-hazards analysis was performed to check strength and association between the determinants and time for SCC.

**Results:**

Out of 252 MDR-TB patients enrolled, sputum culture conversion was observed in 76.6% of the patients by the end of six months. While, 19.0% of the patients failed to achieve negative culture and remained positive after interim report of their treatment. Age > 45 years (HR = 15.22; 95% CI: 7.27-31.83; p<0.001), female gender (6.22; 2.90-13.36; p<0.001), BMI < 18.5 kg/m^2^ (10.28; 5.25-20.11; p<0.001), weight loss (0.03; 0.01-0.06; p<0.001), smoking (0.10; 0.05-0.21; p<0.001), diabetes mellitus (0.02; 0.00-0.04 p<0.001) and disease severity on chest X-ray (CXR) (0.03; 0.01-0.09; p<0.001) were the significant determinants of delayed sputum culture conversion.

**Conclusion:**

MDR-TB patients with older age, low BMI, weight loss, diabetes, smokers and those with disease severity on CXR are less likely to respond to treatment as they displayed delayed SCC. Therefore, such patients should be meticulously followed up for successful management.

## INTRODUCTION

Tuberculosis (TB) is an infectious disease striking all the efforts for better health throughout the world from ancient times and still prevails.^1^ It is one of the world’s most widespread and deadly illnesses caused by inhaled airborne droplet nuclei containing viable organisms, characterized by slowly progressive constitutional symptoms of malaise, anorexia, weight loss, fever, night sweats and often presents with chronic cough and blood-streaked sputum. According to the Centre for Disease Control & Prevention (CDC), the incidence rate of TB has declined by an average of 2% to 3% annually, i.e. from 2.7 during 2019 to 2.2 per 100,000 persons in 2020.^2^ Still, this deadly disease accounts for 1.6 million deaths per year, which is substantially contributed by *human immunodeficiency virus* (HIV)/Acquired *immunodeficiency* syndrome (AIDS).^3^ Pakistan ranks fourth among the 27 low to middle-income countries with the highest TB/MDR-TB burden.^4^ *According to National TB control program, the reported mortalities among TB patients is 34 per 100,000 population per year.^5^* This death rate, despite seeking medical care, reflects the inadequacy of the healthcare system.^6^

TB resurges in the form of drug-resistant TB (DR-TB), which is challenging to treat as its treatment consists of second-line drugs (SLDs) with less efficacy and comparatively more side effects. MDR-TB is the most prevalent type of DR-TB; it has defied all TB control programs globally. The treatment involves an intensive and continuation phase; in the intensive phase, the injectable is added to the SLDs. Its duration may span around eight months, followed by the continuation phase (without injection).^7^ An important indicator of this shift from one phase to another is sputum culture conversion (SCC). Sputum Culture conversion is defined as two consecutive negative sputum cultures with samples collected at least 30 days apart.^8^ Reducing the sputum culture conversion time is an important infection control measure because a culture-positive patients is more likely to transmit MDR-TB.

According to the guidelines, during the intensive phase of treatment, sputum smear microscopy, sputum smear culture and drug-susceptibility testing (DST) should be performed at baseline. While, sputum smear microscopy and sputum culture must be considered every month during follow-up treatment. During the continuation phase, sputum smear microscopy is to be performed monthly on every follow-up visit, whereas sputum culture has to be performed bi-monthly.^9^,^10^ Despite the efforts, the unsuccessful treatment outcomes are evident, and it not only interrupts the disease control agenda but also increases the burden on healthcare finances.^9^,^11^

In addition, factors such as the category of MDR-TB, co-infection with HIV, presence of radiological findings, chronic diseases, therapeutic delay (> 1 month), the quantity of active drugs consumed and drug resistant at treatment initiation delays the time to SCC.^10^-^13^ Delayed SCC results in higher case fatality rates in response to drug toxicity, leading to Extensive Drug-Resistant TB (XDR-TB).^12^ Despite known, the studies on time to SCC are very limited in Pakistan. Therefore, this hospital-based study intended to determine the time for SCC and its determinants among the enrolled MDR-TB patients.

## METHODS

A cross-sectional hospital-based study was conducted at the Programmatic Management of Drug-Resistant TB (PMDT) Unit of Lady Reading Hospital (LRH) in Peshawar, Pakistan, from January 2019 to January 2020. All MDR-TB patients who were culture and smear-positive at the start of the treatment were included in the study. While negative culture patients and those who were culture positive but failed to follow-up (in < 2 months) were excluded from the study. A total of 252 patients fulfilling the inclusion criteria were recruited via the non-probability convenience sampling technique.

Sputum smear microscopy, sputum culture, and chest radiographs (CXR) were obtained at baseline and then at monthly intervals during the intensive phase of treatment, whereas bimonthly in the continuation phase. All patients were tested for blood investigations at baseline, including HIV and then every month as per national DR-TB guidelines. Time to SCC was calculated from the initiation of treatment till the patient had two consecutive negative cultures.

Dependent variables included monthly sputum culture results and time to culture conversion. While socio-demographic characteristics (age, gender, educational status of patients, Body Mass Index), history of previous TB disease (previous treatment category, treatment outcomes and family contacts), presence of any other comorbid disease (diabetes mellitus, HIV AIDS and Hepatitis A, B, C), symptoms at baseline (cough, sputum with cough, blood in sputum, duration of illness, weight loss and night sweating), and clinical characteristics (chest X-Ray findings, and different laboratory findings) were the independent variables.

### Data collection and analysis:

The data were collected using a structured questionnaire and exported to SPSS version 20.0 for statistical analysis. For the study purpose, the descriptive statistic was used for the continuous variables like age and family size, while frequency and percentage was used to display the categorical data like age groups, levels of education, district-wise distribution of residence, family size category, employment status, and occupational status. The Cox proportional-hazards analysis was performed to evaluate the hazard ratio (HR), where the hazard ratio with 95% confidence level was used to report the strength and presence of an association. A p <0.05 was considered statistically significant.

### Ethical Consideration:

The ethics committee of IREB LRH/MTI approved this study [186/30-07-2018]. The patients were informed regarding the purpose of the study and written informed consent was obtained from the patients or caretakers before inclusion.

## RESULTS

In this study, 252 MDR-TB patients were included from different areas of Khyber Pakhtunkhwa, the Ex-FATA region and Afghanistan. The mean age of these patients was 31.97 ± 15.34 years. The distribution of patients by age showed that most of the patients (76.2%) were between 21 to 30 years of age. More than half of them were females (56.0%) and underweight (67.1%) (Table-I). The majority of patients (94.4%) belonged to poor families with monthly incomes up to 15,000 PKR.

**Table-I:** Baseline characteristics of the enrolled MDR-TB patients (n= 252).

Variables			n(%)
Gender		Male	111(44.0)
		Female	141(56.0)
Age Groups (years)		≤ 10	01(0.4)
		11 – 20	61(24.2)
		21 – 30	90(35.7)
		31 – 40	40(15.9)
		41 – 50	21(8.3)
		51 – 60	22(8.7)
		> 61	17(6.7)
BMI (kg/m^2^)		< 18.5 kg/m^2^	169(67.1)
		≥ 18.5 kg/m^2^	83(3.9)
Marital Status		Single	152(60.3)
		Married	100(39.7)
Educational Status		Illiterate	196(77.7)
		Educated	56(22.2)
Smoking		Yes	104(41.26)
		No	148(58.73)
Diabetic Mellitus		Yes	63(25)
		No	190(75.3)
Symptoms	Cough Duration	< 14 days	123(48.8)
		≥ 14 days	129(51.19)
	Weight Loss	Yes	67(26.58)
		No	185(73.4)
Disease severity on CXR		Yes	95(37.69)
		No	157(62.30)
Monthly Income		≤ 5,000 PKR	37(14.7)
		6,000-10,000 PKR	175(69.4)
		11,000-15,000 PKR	26(10.3)
		16,000-20,000 PKR	02(0.8)
		21,000-25,000 PKR	07(2.8)
		26-30	03(1.2)
		≥ 30	02(0.8)
No. of rooms in patients house		≤ 4	193(76.6)
		5 – 9	39(15.5)
		≥ 10	20(7.9)
No. of persons living with the patient in the household		≤ 5	22(8.7)
		6-10	93(36.9)
		11-15	71(28.2)
		16-20	33(13.1)
		21-25	13(5.2)
		26-30	09(3.6)
		31-35	01(0.4)
		36-40	03(1.2)
		≥ 41	07(2.7)
Registration Group		New	27(10.7)
		Cat I Failure	53(21.0)
		Cat II Failure	117(46.4)
		Relapse of Cat I	21(8.3)
		Relapse of Cat II	24(9.5)
		Default of Cat I	9(3.6)
		Default of Cat II	1(0.4)
Any close contact with patients of DS-TB in family		Yes	151(59.9)
		No	101(40.1)
Any close contact with patients of MDR-TB in family		Yes	12(4.8)
		No	240(95.2)
Registration with DOTS center		Registered	210(83.3)
		Not Registered	42(16.7)

DS-TB: Drug-sensitive TB; MDR-TB: Multidrug-Resistant Tuberculosis; DOTS: Directly Observed Therapy

Furthermore, most of them were living in small and overcrowded housing. Among the enrolled patients, 3(1.2%) showed reactive status to HIV-AIDS along with MDR-TB. Study cases belonged to different registration groups; most (46.4%) were from Cat II Failure. Of the total registered patients at the DOTS center, 190(75.4%) were registered with the DOTS center located at any government hospital registered with NTP (National TB Program) and remaining 62(24.6%) were registered with Public-Private Mix (PPM) center for TB treatment.

**Fig.1:**
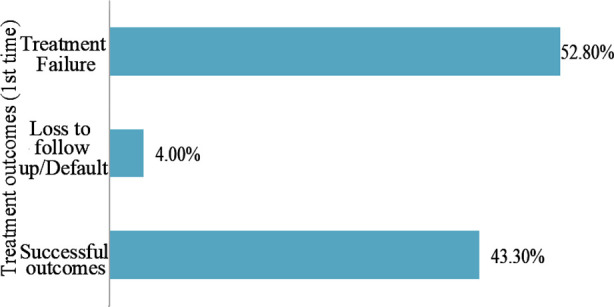
Treatment outcomes among MDR-TB patients treated for the first time at the study site.

Most of the patients (52.8%) were declared having treatment failure after the first time treatment as shown in Fig.1. The results of drug susceptibility test (DST) of these patients at baseline of their treatment showed that out of the 252 *M. tuberculosis* isolates tested for drug sensitivity against individual drugs, 100% strains were found to be sensitive to Rifampicin and Isoniazid, followed by Pyrazinamide (91.3%), Ofloxacin (64.7%), Ethambutol (44.4%), Streptomycin (42.5%), Ethionamide (11.9%), Capreomycin (11.5%), Amikacin (4.0%) and Kanamycin (3.6%).

**Fig.2:**
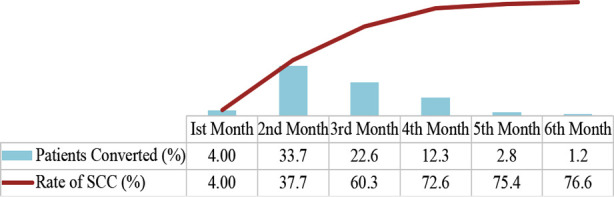
Monthly sputum culture conversion and its rate over time.

Out of 252 patients with sputum culture-positive at baseline, about 76.6% achieved culture conversion by the end of study duration (6^th^ months), whereas 19.0% of the patients did not have culture conversion and remained positive after interim report of their treatment. The mortality rate was 4.4% during the overall study time (Fig.2).

In univariate analysis, we found that factors associated with reduced rate of sputum culture conversion were age > 45 years (HR = 15.22; 95% CI: 7.27-31.83; p<0.001), female gender (6.22; 2.90-13.36; p<0.001), BMI < 18.5 kg/m^2^ (10.28; 5.25-20.11; p<0.001), weight loss (0.03; 0.01-0.06; p<0.001), smoking (0.10; 0.05-0.21; p<0.001), diabetes mellitus (0.02; 0.00-0.04 p<0.001) and disease severity on CXR (0.03; 0.01-0.09; p<0.001) (Table-II).

**Table-II:** Factors associated with reduced rate of sputum culture conversion.

Variables		Culture Conversion		HR (95% CI)	p-value

		Yes	No		
Age Group	≤ 45 years	150	11	Ref	< 0.001*
	> 45 years	43	48	15.22(7.27-31.83)	
Gender	Male	102	09	Ref	< 0.001*
	Female	91	50	6.22(2.90-13.36)	
Marital Status	Single	125	27	Ref	0.009*
	Married	68	32	2.17(1.20-3.93)	
No. of rooms in patients house	≤ 4	150	49	Ref	0.379
	> 4	43	19	0.71(0.33-1.52)	
BMI	≥ 18.5 kg/m^2^	40	43	Ref	<0.001*
	< 18.5 kg/m^2^	153	16	10.28(5.25-20.11)	
Education Status	Illiterate	150	46	Ref	0.968
	Educated	39	17	0.98(0.48-1.99)	
Cough Duration	< 14 days	110	13	Ref	0.987
	≥ 14 days	73	56	4.69(2.38-9.24)	
Weight Loss	No	173	12	Ref	<0.001*
	Yes	21	46	0.03(0.01-0.06)	
Smoking	No	136	12	Ref	<0.001*
	Yes	57	47	0.10(0.05-0.21)	
Diabetic Mellitus	No	178	12	Ref	< 0.001*
	Yes	11	52	0.02(0.00-0.04)	
Disease severity on CXR	No	150	07	Ref	< 0.001*
	Yes	38	57	0.03(0.01-0.09)	

Data is presented as frequencies,

* p < 0.05 is considered significant.

## DISCUSSION

This study focuses on the determination of time for SCC and its determinants for controlling MDR-TB in KPK. As Pakistan is among the countries with the highest MDR-TB burden and restricted resources, the disease is treated under programmatic management of drug-resistant at various specified sites in different areas of the country. With such great efforts, the overall successful treatment outcome rate among MDR-TB patients is around 75%, as indicated by different local studies.^13^-^17^

In the present study, 76.6% of MDR-TB patients treated at PMDT LRH achieved culture-negative results within the average of 75 days (30 – 180 days) of the treatment. These findings are somewhat similar to that reported in Lativa^18^ and Ethiopia^19^, in which 77% and 86.7% of patients achieved culture conversion within 60 to 65 days, respectively. In contrast, a study from China showed that 76.3% of the patients achieved culture negative status in an average of 92 days.^20^ A large-scale study including the data from five different countries, i.e. Peru, Latvia, Estonia, Russia and Philippines, from 2000 to 2004, showed that 85.4% of the enrolled TB patients had SCC to negative in an average of 90 days.^12^ A local Pakistani study also reported prolong median conversion time i.e. 191 days.^21^ The possible explanation for these differences might be the variation in defining the outcome variables and sample size of these studies.^19^ In the present study, the time for culture conversion was defined by two consecutive negative culture results, whereas in the comparative studies, it was defined by five consecutive negative culture results. Furthermore, the strict programmatic system of treatment was followed for the selection of individualized treatment regimen for each patient according to their drug susceptibility testing against MTB isolates and past treatment, monthly visits with trained treatment supporters for daily DOT at home, strict monitoring on the phone from time to time, monthly counselling by a trained psychologist, home visits by treatment coordinators at baseline and after every six months of treatment and emergency visit in case of any delay or missed appointment. With all these practices, the patients mostly became compliant with the treatment, and only 40 of them lost to follow-up at the specific study site.

But we still lack to manage the delayed culture conversion; 60.3% of patients achieved negative culture conversion in the first three months while 16.4% of cultures converted later after their 3^rd^ month of treatment. By the end, 19.0% remained positive, and the mortality rate was 4.4%. Most of the patients (52.8%) were declared having treatment failure after the first time treatment, while 43.3% had successful outcomes. A Chinese study reported successful outcomes among 60.4% of the MDR-TB patients, and 39.6% of them either experienced treatment failure or died.^20^

The factors responsible for culture conversion were also studied, age > 45 years, female gender, BMI < 18.5 kg/m^2^, weight loss, smoking, diabetes mellitus and disease severity on CXR a significant role in culture conversion and were associated with delayed SCC (p<0.001). In contrast, similar studies from China reported no significant association between older age, female gender and culture conversion (p>0.05)^20^. Furthermore, a systematic review of literature also suggested that no particular gender is at a higher risk of acquiring MDR-TB results^22^. But our findings are consistent with an Indonesian study presenting significant impact of female gender on the SCC^23^. Other factors like severity of disease, including cavitary disease and baseline high bacillus load, showed a significant positive association with delayed SCC. Some other studies also explain these findings.^19^,^24^,^25^ These studies explained that MDR-TB patients with the severe disease having more chest cavities were less responsive to drugs than other normal patients.

SCC is an important indicator for monitoring treatment outcomes among MDR-TB patients. Therefore, in order to avoid treatment failure and ensure compliance, the treatment and management must focus on the patient’s comfort, which could be achieved by more rapid SCC that would ultimately simplify the therapy with reduced duration of injectable drug used. Despite the strengths, certain limitations need to be addressed.

### Limitations of the study:

The study did not evaluate the detailed smoking patterns of differential frequencies among the studied cases. Furthermore, this was a single-center region-specific study with limited sample size; a large-scale multicenter study representing the Pakistani population is required to better explain the SCC influence on treatment outcomes among MDR-TB patients in the country.

## CONCLUSION

Our findings show that the overall success rate (cultures converted to negative) was 76.6% by the end of the third month. Despite programmatic management with closed monitoring and monthly social support incentives, few factors like old age, BMI < 18.5 kg/m^2^, weight loss, smoking, diabetes mellitus and disease severity on CXR affect the timely culture conversion in DR-TB/MDR-TB patients. As mortality occurred in the early few months of treatment, indicating the need for timely diagnosis and treatment initiation without any delay.

### Authors Contribution:

**MAK, ZI:** Are responsible for the concept and study design.

**MAK, MN:** Contributed to the data collection and literature review.

**MAK, AA, MN:** Are responsible for data analysis and interpretation and drafting of the manuscript.

**ZI, AA, MAK:** Contributed to the critical review, revision and final approval of the study.

All the authors are equally responsible and accountable for the accuracy and integrity of the work.

## References

[1] World Health Organization https://www.who.int/news-room/fact-sheets/detail/tuberculosis.

[2] CDC (2020). //www.cdc.gov/tb/statistics/reports/2019/default.htm.

[3] MacNeil A, Glaziou P, Sismanidis C, Date A, Maloney S, Floyd K (2020). Global epidemiology of tuberculosis and progress toward meeting global targets-worldwide, 2018. Morbidity and Mortality Weekly Report.

[4] World Health Organization http://www.emro.who.int/tuberculosis/epidemiological-situation/epidemiological-situation.html.

[5] National Institute Of Health Islamic Republic of Pakistan. National TB Control Program.

[6] Pai M, Temesgen Z (2019). Quality:The missing ingredient in TB care and control. Clin Tuberc Other Mycobact Dis.

[7] Nahid P, Mase SR, Migliori GB, Sotgiu G, Bothamley GH, Brozek JL (2019). Treatment of drug-resistant tuberculosis. An official ATS/CDC/ERS/IDSA clinical practice guideline. Am J Respir Crit Care Med.

[8] Kurbatova EV, Cegielski JP, Lienhardt C, Akksilp R, Bayona J, Becerra MC (2015). Sputum culture conversion as a prognostic marker for end-of-treatment outcome in patients with multidrug-resistant tuberculosis:A secondary analysis of data from two observational cohort studies. Lancet Respir Med.

[9] Khan MA, Mehreen S, Basit A, Khan RA, Jan F, Ullah I (2015). Characteristics and treatment outcomes of patients with multi-drug resistant tuberculosis at a tertiary care hospital in Peshawar, Pakistan. Saudi Med J.

[10] Pontali E, Matteelli A, Migliori GB (2013). Drug-resistant tuberculosis. Curr Opin Pulm Med.

[11] Khan S, Khan MA, Nasir SM, Naveed A, Latif A, Javaid A (2019). Factors associated with new and re-treated Multidrug-Resistant Tuberculosis in Khyber Pakhtunkhwa. Pak J Chest Med.

[12] Kurbatova E, Gammino V, Bayona J, Becerra M, Danilovitz M, Falzon D (2012). Predictors of sputum culture conversion among patients treated for multidrug-resistant tuberculosis. Int J Tuberc Lung Dis.

[13] Khan MA, Mehreen S, Basit A, Khan RA, Javaid A (2015). Predictors of poor outcomes among patients treated for multidrug-resistant tuberculosis at Tertiary Care Hospital in Pakistan. American-Eurasian J Toxicol Sci.

[14] Rao NA, Mahfooz Z, Irfan M (2009). Treatment outcome of multi-drug resistant tuberculosis in a tertiary care hospital in Karachi. J Pak Med Assoc.

[15] Javaid A, Ullah I, Masud H, Basit A, Ahmad W, Butt ZA (2018). Predictors of poor treatment outcomes in multidrug-resistant tuberculosis patients:a retrospective cohort study. Clin Microbiol Infect.

[16] Liu CH, Li L, Chen Z, Wang Q, Hu YL, Zhu B (2011). Characteristics and treatment outcomes of patients with MDR and XDR tuberculosis in a TB referral hospital in Beijing:a 13-year experience. PloS one.

[17] Batool R, Imran M, Kandhro AH, Salahuddin N, Uddin MKH (2017). Resistance Patterns among Multidrug-Resistant Tuberculosis Patients:A Multi-Center Study from Pakistan. IJEHSR.

[18] Holtz TH, Sternberg M, Kammerer S, Laserson KF, Riekstina V, Zarovska E (2006). Time to sputum culture conversion in multidrug-resistant tuberculosis:predictors and relationship to treatment outcome. Ann Intern Med.

[19] Yihunie Akalu T, Muchie KF, Alemu Gelaye K (2018). Time to sputum culture conversion and its determinants among Multi-drug resistant Tuberculosis patients at public hospitals of the Amhara Regional State:A multicenter retrospective follow up study. PLoS One.

[20] Liu Q, Lu P, Martinez L, Yang H, Lu W, Ding X (2018). Factors affecting time to sputum culture conversion and treatment outcome of patients with multidrug-resistant tuberculosis in China. BMC Inf Dis.

[21] Qazi F, Khan U, Khowaja S, Javaid M, Ahmed A, Salahuddin N (2011). Predictors of delayed culture conversion in patients treated for multidrug-resistant tuberculosis in Pakistan. Int J Tuberc Lung Dis.

[22] Faustini A, Hall AJ, Perucci CA (2006). Risk factors for multidrug resistant tuberculosis in Europe:A Systematic Review. Thorax.

[23] Putri FA, Burhan E, Nawas A, Soepandi PZ, Sutoyo DK, Agustin H (2014). Body mass index predictive of sputum culture conversion among MDR-TB patients in Indonesia. Int J Tuberc Lung Dis.

[24] Basit A, Ahmad N, Khan AH, Javaid A, Syed Sulaiman SA, Afridi AK (2014). Predictors of two months culture conversion in multidrug-resistant tuberculosis:Findings from a retrospective cohort study. PLoS One.

[25] Mota PC, Carvalho A, Valente I, Braga R, Duarte R (2012). Predictors of delayed sputum smear and culture conversion among a Portuguese population with pulmonary tuberculosis. Revista Portuguesa de Pneumologia (English Edition).

